# Differential accumulation of proteins in oil palms affected by fatal yellowing disease

**DOI:** 10.1371/journal.pone.0195538

**Published:** 2018-04-05

**Authors:** Sidney Vasconcelos do Nascimento, Marcelo Murad Magalhães, Roberto Lisboa Cunha, Paulo Henrique de Oliveira Costa, Ronnie Cley de Oliveira Alves, Guilherme Corrêa de Oliveira, Rafael Borges da Silva Valadares

**Affiliations:** 1 Instituto Tecnológico Vale, Belém, Pará, Brazil; 2 Programa de Pós-Graduação em Biotecnologia Aplicada à Agropecuária, Universidade Federal Rural da Amazônia, Belém, Pará, Brazil; 3 Analysis of sustainable system laboratory, Embrapa Amazônia Oriental, Belém, Pará, Brazil; Bhabha Atomic Research Centre, INDIA

## Abstract

There is still no consensus on the true origin of fatal yellowing, one of the most important diseases affecting oil palm (*Elaeis guineensis* Jacq.) plantations. This study involved two-dimensional liquid chromatography coupled with tandem mass spectrometry (2D-UPLC-MS^E^) analyses to identify changes in protein profiles of oil palms affected by FY disease. Oil palm roots were sampled from two growing areas. Differential accumulation of proteins was assessed by comparing plants with and without symptoms and between plants at different stages of FY development. Most of the proteins identified with differential accumulation were those related to stress response and energy metabolism. The latter proteins include the enzymes alcohol dehydrogenase and aldehyde dehydrogenase, related to alcohol fermentation, which were identified in plants with and without symptoms. The presence of these enzymes suggests an anaerobic condition before or during FY. Transketolase, isoflavone reductase, cinnamyl alcohol dehydrogenase, caffeic acid 3-O-methyltransferase, S-adenosylmethionine synthase, aldehyde dehydrogenase and ferritin, among others, were identified as potential marker proteins and could be used to guide selection of FY-tolerant oil palm genotypes or to understand the source of this anomaly. When comparing different stages of FY, we observed high accumulation of alcohol dehydrogenase and other abiotic stress related-proteins at all disease stages. On the other hand, biological stress-related proteins were more accumulated at later stages of the disease. These results suggest that changes in abiotic factors can trigger FY development, creating conditions for the establishment of opportunistic pathogens.

## 1. Introduction

The main oil source of plant origin in the world is the oil palm (*Elaeis guineensis* Jacq.). This species has large economic and social importance in producing countries. The fruit of this palm species contains palm oil and palm kernel oil, used in processed foods, pharmaceuticals and cosmetics, as well as for sustainable energy generation [1, 2].

The first reports of FY date to the 1980s, and its etiology remains unknown. A good deal of research has been done to understand FY’s cause, considering biotic factors [3, 4, 5, 6, 7, 8, 9], but there is still no consensus on the true origin of this anomaly. The current trend is to focus on abiotic factors, mainly associated with water balance distribution [10, 11, 12, 13], as well as the limitation of drainage, soil nutrition and oil palm root system [14, 15].

Due to inadequate soil management, growing areas suffer from soil compaction, reducing drainage and physical impedance, associated with long flooded periods (up to six months). In these conditions, it is impossible for the plants to maintain their regular metabolic activities, because in waterlogged conditions the root system cannot properly metabolize energy and suffers from fermentation [16, 17]. Consequently, anaerobic metabolism triggers an increase in glycolysis, increasing gene transcription of enzymes related to ethanol fermentation. In addition to these alterations, carbohydrate metabolism produces more substrates for fermentation [17]. Concomitantly, the activity of the antioxidant system increases, and in the final stages, opportunistic pathogens attack the roots [18].

In this context, it is very important to identify alterations at the molecular level in plants with FY versus healthy ones. This can shed light on the tolerance mechanism associated with this problem. In this respect, proteomic techniques enable obtaining a protein profile with precision and sensitivity with the help of mass spectrometry and bioinformatics tools. These techniques have been used to analyze plant responses to different environmental conditions, including soil flooding [19, 20, 21]. In a recent work, Vargas et al. (2016) [22] established a protocol for analysis of metabolites in oil palm leaves, which can contribute to the identification of biochemical markers for FY. In addition, techniques in proteomics should help improve the knowledge about metabolic changes related to FY tolerance or development.

Our hypothesis is that the abiotic factors can favor the start of FY and this problem can be aggravated by biological agents during its development. The objective of this study was to obtain the proteome differential of plants with and without apparent FY symptoms, to identify proteins related with the tolerance, start and/or development of FY in oil palms.

## 2. Material and methods

### 2.1. Plant material

Oil palm roots were sampled in field conditions of two areas in August 2016 (after a period of higher rainfall, when the incidence of FY in the field is greater), in a sandy yellow dystrophic latosol in the municipality of Mojú, Pará state, in northern Brazil (1°26’S and 48°26’W, 21 m above sea level). One area belongs to the company Marborges Agroindústria S.A. (area I) and another to the company Biopalma (area II). Sampling was carried out at Marborges S.A and Biopalma S.A farms with their logistic support, safety instructions and authorizations. No specific permission were required for these locations/activities and the study did not involve endangered or protected species. The region has tropical climate with mean annual temperature of 25°C and average rainfall 2,319 mm, mainly distributed from January to August. The plants of area I are progenies of Deli x Lamé of planting dated of 2000. Plants of area II are progenies of Deli x Nigeria and the planting date of 2010. The plants were cultivated in full sunlight with spacing of 9.0 x 9.0 m. The standard crop management was performed in relation to soil nutrition and control of pathogens and insects. Irrigation was not necessary due to the abundant rainfall during the entire crop development. Asymptomatic plants and plants with symptoms in the initial, intermediate and late stages of FY symptoms were collected according to the classification proposed by Souza et al. (2000) [23].

The roots were collected 1 m from a stipe basis in a hole with and 50 x 50 x 20 cm length, width and depth, respectively. After washing with water, the roots were kept in liquid nitrogen and transported to the laboratory of Instituto Tecnológico Vale.

For comparisons between plant proteomes with and without FY symptoms, roots from fifteen plants were pooled in order to obtain three biological replicates for each condition, each replicate consisting of roots from five plants. For the proteomic analyzes between the different stages of FY development, roots from five plants were pooled to obtain one sample for each stage. Three analytical replicates (LC-MS runs) were obtained from each sample.

### 2.2. Protein extraction and quantification

Proteins were isolated following the SDS (sodium dodecyl sulfate)/phenol protocol proposed by Wang (2006) [24] with some modifications (S1 Table). The protein concentration of each sample was measured on the Qubit 2.0 fluorometer (Invitrogen, Thermo Fisher Scientific), using Qubit protein assay kit according to the manufacturer's protocol.

### 2.3. Protein digestion

For protein digestion, 50 μg of proteins from each sample were treated with 5 mM of dithiothreitol (DTT) for 25 minutes at 56°C and then with 14 mM of iodoacetamide (IAA) for 30 minutes at room temperature. Then residual quenching of the IAA was performed by adding 5 mM of DTT for 15 minutes at room temperature. After 1/5 (v/v) dilution of the samples, ammonium bicarbonate (50 mM) was added to CaCl_2_ (1 mM) for all the samples, followed by addition of 20 ng/μL of trypsin (Trypsin Gold, Promega, WI, USA). The samples were left for digestion for 16 hours at 37°C. The enzymatic reaction was stopped by adding 0.4% trifluoroacetic acid (TFA).

### 2.4. Protein desalting

Samples were desalted using a C_18_ Sep-Pack column (Oasis) for solid-phase extraction. The column was conditioned with 3 mL of 100% acetonitrile (ACN); equilibrated with 1 mL of 50% ACN 50%/0.1% formic acid and then 0.1% TFA (3 mL). The samples were loaded into the column and washed with 3 mL of 0.1% TFA; equilibrated with 0.1% formic acid (1 mL). The samples were then eluted with, in order, 50% ACN/0.1% formic acid (2 mL) and 80% ACN/0.1% formic acid (1 mL), followed by drying in a vacuum concentrator and resuspension using 50 μL of ammonium formate 10 mM, before UPLC-MS injection.

### 2.5. 2D-UPLC- mass spectrometric analysis

An aliquot containing (4.5 μg of each sample was loaded for separation into an Nano Acquity UPLC^®^ System (Waters Corp.) equipped with 2D online dilution technology. The first chromatographic dimension of the peptide fraction was ascertained under basic (pH = 10) conditions in a BEH C_18_ 300 Å, 5 μM 300 um x 50 mm reverse phase column (XBridge^TM^, Waters Corp.). This was performed at a flow rate of 2 μL/min. Eluent A was aqueous 20 mM FA (pH = 10) and eluent B was neat ACN. All samples were analyzed using a five-step fractionation method. The fractions were eluted from the first dimension using a composition of 10.8, 14.0, 16.7, 20.4 or 65% of eluent B, respectively.

The fractionation process was programmed to start immediately after completion of sample loading (20 min at 10 μL/min with 3% B). Each first dimension elution step was performed with 20 min run time using a flow rate of 2 μL/min. Eluent peptide was mixed online with 10 μL/min of 0.1% TFA solution (1:10 dilution) before being trapped in the trapping column (100 μm x 100 mm), packed with 1.7 μm 100 Å silica-based C_18_ (Symmetry, Waters Corp, Milford, MA).

The mobile phase for the second chromatographic dimension (low pH RS) was 0.1% FA in water (immobile phase A) and 0.1% FA in ACN (mobile phase B). The second dimension column was 100 μm x 10 mm C_18_ packed with changed surface hybrid (CSH) 1.8 mm particles (Acquity UPLC M-Class CSH C_18_, Waters Corp., Milford, MA). The flow rate for the second dimension separation was 400 nL.min-1, while the column was maintained at 55°C. A 40-minute gradient from 3 to 40% B was used to separate peptides in the second separation dimension. The column was then washed using 90% B for 1 minute and equilibrated with 3% B for 7 minutes before returning to the next of fractionation.

Mass spectra were obtained with a Synapt G2-S spectrometer equipped with standard electrospray ionization (ESI) source (Waters). For all measurements, the mass spectrometer was operated in positive ion resolution mode. Mass spectra were acquired in continuum mode over an *m/z* range of 50–1200, using a capillary voltage of 2.6 KV, source temperature of 100°C, source offset voltage of 100 V, cone gas flow of 50 L/h and cone voltage of 40 V. The spectral acquisition time at each energy setting was 0.5 seconds. A solution of 0.2 μM Glu^1^-fibrinopeptide (785.8427 Da) was used as a lock-mass solution, delivered at a flow rate of 0.5 μL/min using an auxiliary pump of the liquid chromatography system. The lock-mass was sampled every 30 sec using 0.1 second scans over the same mass range.

### 2.4. Experimental design and data analysis

We compared asymptomatic and symptomatic roots of plants collected in two different areas. Datasets were analyzed separately. A comparison was also made between proteomes obtained from roots in the initial, intermediate and advanced stages (stages 1, 5 and 9, respectively) in order to identify differential accumulation throughout the progression of FY symptoms. This last experiment was performed only with the proteomes of roots of plants sampled in the first area.

The peptic identification list was generated by the Protein Lynx Global Server (PLGS) 3.0.2 (Waters Corp, Milford, MA, USA) using a combination of exact mass and MS^E^ fragment data. Processed spectra were then searched against a custom protein database compiled from *Elaeis guineensis* Jacq. at the website of the National Center for Biotechnology Information (NCBI:, 04/2016). Management and validation of mass spectrometry data were performed using the Scaffold Q+ (Scaffold version 4.5.1, Proteome Software Inc., Portland, OR). Protein identification was only accepted if the peptide identification probability was greater than 90% and proteins greater 95% accordingly to the peptideprophet and proteinprophet algorithms [25]. Differentially expressed proteins were determined by applying a permutation test with significance level grater than 95% (*p* < 0.05). Statistical significance (*P*-values) for quantitative measurements are available in S2A, S3A and S4A Tables. With few exceptions, in this study we used the cutoff criterion of more or less abundant proteins of log_2_ fold change ≥ 1 for more abundant proteins and log_2_ fold change ≤ -1 for less abundant proteins. In addition, we highlighted proteins identified in at least two replicates. Functional annotation of proteins was performed with Blast2GO version 4.0 (Biobam). The heatmap with proteins involved in stress response and energy metabolism was calculated by the R statistical software, through the utilization of the *heatmap*.*2* function available in the *gplots* R package.

## 3. Results

### 3.1. Oil palm root protein profile from FY occurrence areas

**Fig 1:**
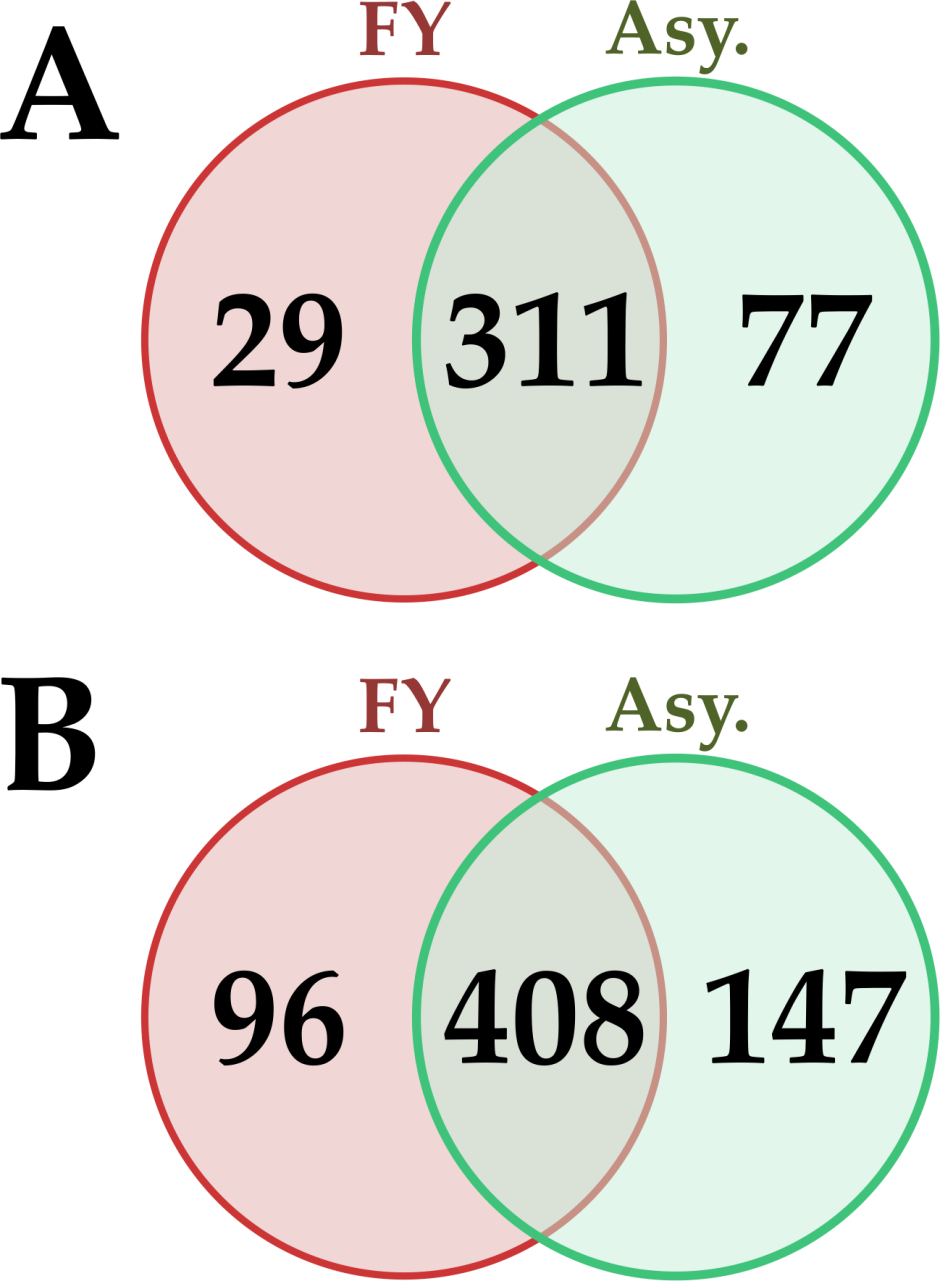
Venn diagram of proteins identified in oil palm roots of plants with symptoms (FY.), asymptomatic (Asy.), and in both conditions (intersection). (**A**) Proteins from plants sampled in area I. (**B**) Proteins from plant sampled from area II.

All told, 417 and 651 proteins were identified and quantified in roots of oil palms sampled from areas I and II, respectively. The set of proteins presented some distinctions between the plants of the two areas. All the proteins identified in this study are detailed in S2, S3 and S4 Tables. Proteomic data distribution is displayed in the Venn diagram (Fig 1).

Comparing plant protein profiles with and without FY symptoms, 127 proteins were up-regulated and 162 were down-regulated in plants with FY symptoms. In plants of area II, 179 and 239 proteins were up- and down-regulated, respectively.

Among the most differentially accumulated proteins present in the current dataset are those involved in the production of energy and proteins related to different mechanisms of stress response.

### 3.2. Proteins related to biotic and abiotic stresses

In plants of area I, several proteins related to stress responses were differentially abundant (Table 1).

**Table 1:** Differentially abundant proteins directly or indirectly related to stress response in oil palm roots of area I with log_2_ fold change ≥ 1 or ≤ -1.

Protein name	MW^a^ (kDa)	Accession	Log_2_ FC^b^
Hypersensitive-induced response protein 1	31	gi|743755084	FY^c^
Nucleoside diphosphate kinase B-like	17	gi|743827402	FY
Nucleoside diphosphate kinase B	16	gi|743778585	FY
Glucan endo-1,3-beta-glucosidase-like	37	gi|743785400	6.51
Patellin-3-like	54	gi|743816880	5.77*
Patellin-3-like	61	gi|743764635	4.35
Hypersensitive-induced response protein 1 X2	31	gi|743798950	2.32
22.0 kDa class IV heat shock protein-like	22	gi|743765011	2.22
Acidic endochitinase-like	31	gi|743796702	2.14
Hydroxyacylglutathione hydrolase cytoplasmic	29	gi|743891582	1.92
Apyrase 2	50	gi|743789264	1.91
14-3-3-like protein D isoform X1	30	gi|743757050	1.69
Chaperone protein ClpB1	101	gi|743756256	1.57
Guanine nucleotide-binding protein subunit beta-like protein A	36	gi|743794305	1.51
Formamidase C869.04	50	gi|743761007	1.42
Peroxiredoxin	17	gi|192910922	1.29
Leucine aminopeptidase 2, chloroplastic-like	56	gi|743857317	1.29
Glycine-rich RNA-binding protein	16	gi|648174145	1.22
Beta-1,3-glucanase	36	gi|192910884	1.2
Flavonoid 3',5'-methyltransferase-like	27	gi|743813658	1.18
Caffeoyl-CoA O-methyltransferase-like isoform X1	22	gi|743813662	1.14
18.1 kDa class I heat shock protein	18	gi|743810653	1.14
Uncharacterized protein phloem protein 2-like A4-like	20	gi|743855845	1.12
Guanine nucleotide-binding protein subunit beta-like protein A	35	gi|743772066	-1.25
Thaumatin-like protein 1b	25	gi|743826113	-1.26
Oryzain alpha chain-like	51	gi|743805669	-1.27
Lipoxygenase homology domain-containing protein 1-like	19	gi|743778359	-1.31
Superoxide dismutase [Cu-Zn], chloroplastic	23	gi|743852970	-1.38
Pathogenesis-related protein	18	gi|192910872	-1.44
Glucan endo-1,3-beta-glucosidase-like	36	gi|743875101	-1.53
Annexin D1-like	36	gi|743849454	-1.75*
Glutathione S-transferase	24	gi|448872672	-1.85
Universal stress protein A-like protein	24	gi|743845102	-2.05
Membrane steroid-binding protein 2	29	gi|743776234	-2.22
Aspartic proteinase oryzasin-1-like	59	gi|743794899	-2.5
Osmotin-like protein	28	gi|743775988	-3.26
Aspartic protease in guard cell 1-like	48	gi|743766057	Asy^d^.
Profilin 2	14	gi|192910850	Asy.
Pathogenesis-related protein PRB1-2-like	23	gi|743761748	Asy.
Fumarilacetoacetase	47	gi|743767417	Asy.
20 kDa chaperonin, chloroplastic-like	27	gi|743774176	Asy.
Peptidyl-prolyl cis-trans isomerase FKBP12 isoform X1	12	gi|743849924	Asy.
Universal stress protein A-like protein	18	gi|743773844	Asy.*
Mannose/glucose-specific lectin-like isoform X2	21	gi|743759608	Asy.*
Pathogenesis-related protein PR-4-like	15	gi|743774487	Asy.*
Subtilisin-like protease	81	gi|743774266	Asy.*
Ferritin-4, chloroplastic-like	29	gi|743873486	Asy.*
L-ascorbate oxidase homolog	61	gi|743793209	Asy.
Peroxidase 17-like	39	gi|743840871	Asy.
L-ascorbate peroxidase 6, chloroplastic	39	gi|743816733	Asy.
Peroxidase 12-like, partial	22	gi|743763659	Asy.
Superoxide dismutase [Cu-Zn]	15	gi|743845883	Asy.
L-ascorbate peroxidase, cytosolic-like	28	gi|743787774	Asy.*

^a^MW = Molecular weight.

^b^Log_2_ FC = Log_2_ fold change.

^c^FY = found exclusively in plants with FY symptoms

^d^Asy. = found exclusively in asymptomatic plants.

* Statistically significant at *p* <0.05 available in S2A, S3A and S4A Tables.

In plants of this area, proteins well-known for their importance in plant defense response such as glucan endo-1,3-beta-glucosidase, acidic endochitinase, apyrase 2, flavonoid 3', 5'-methyltransferase, patellins and caffeoyl-CoA O-methyltransferase were up-regulated in plants with FY symptoms. Among proteins with reduced accumulation were thaumatin, lipoxygenases, some pathogenesis-related proteins, annexins, subtilisin and osmotin (Table 1). On the other hand, most of the proteins involved in the response mechanisms of oil palm roots of area I were more accumulated or identified only in asymptomatic plants. Still regarding asymptomatic plants, among the specific proteins identified from the antioxidant system in oil palm roots sampled from area I were L-ascorbate peroxidase, and superoxide dismutase [Cu-Zn].

In plants sampled from area II, we can highlight two proteins (LOC105031936 and LOC105038753) that had similarity (blastp) with sieve element occlusion (accessions gi|743864273 and gi|743765428, NCBI). These proteins were exclusively found in plants with FY symptoms (Table 2). Most of the proteins which were found only in plants with symptoms or that were up-regulated in this condition are related to defense against fungal pathogens. On the other hand, stress-related proteins involved in response against biotic and abiotic stress, such as S-adenosylmethionine synthase, transketolase and isoflavone reductases, among others, and those involved in the antioxidant system, were identified only in asymptomatic plants.

**Table 2:** Differentially abundant proteins directly or indirectly related to the stress response in roots of plants of area II with log_2_ fold change ≥ 1 or ≤ -1.

Protein name	MW^a^ (kDa)	Accession	Log_2_ FC^b^
L-ascorbate peroxidase 4	31	gi|743779328	FY^c^
Nucleoredoxin 1–1	69	gi|743840630	FY
Glutathione S-transferase	25	gi|743792918	FY
Cationic peroxidase 1-like	34	gi|743782272	FY
Glutathione S-transferase F11-like	25	gi|743889616	FY
Cationic peroxidase SPC4-like	24	gi|743817481	FY
Mavicyanin-like	18	gi|743859187	FY
Allene oxide cyclase 1, chloroplastic-like	27	gi|743756470	FY
Alpha carbonic anhydrase 7-like	33	gi|743864471	FY
Chemocyanin-like	13	gi|743800853	FY
Alpha-mannosidase	116	gi|743813312	FY
Ubiquitin-like isoform X2	14	gi|743857302	FY
Serine protease EDA2 isoform X2	50	gi|743777910	FY
Peroxidase 12-like, partial	22	gi|743763659	FY*
Manganese superoxide dismutase	27	gi|406870049	FY
Monodehydroascorbate reductase,chloroplastic X2	55	gi|743854818	FY*
Germin-like protein 5–1	31	gi|743855137	FY*
Pathogenesis-related protein 1-like	17	gi|743844417	FY*
Mannose/glucose-specific lectin-like X1	31	gi|743759606	FY*
Subtilisin-like protease SBT3.5	73	gi|743829002	FY*
Beta-1,3-glucanase	36	gi|192910882	FY*
Protein LOC105031936	81	gi|743864273	FY*
Protein LOC105038753	81	gi|743765428	FY*
Aspartic proteinase oryzasin-1-like	59	gi|743794899	5.81
Peroxiredoxin	17	gi|192910922	4.82
Putative phosphatidylglycerol/phosphatidylinositol transfer protein DDB_G0282179	17	gi|743877681	4.72
Osmotin-like protein	28	gi|743775988	4.15
Caffeoyl-CoA O-methyltransferase-like	38	gi|743813686	3.57
Pathogenesis-related protein PRB1-2-like	24	gi|743761746	3.5
L-ascorbate peroxidase, cytosolic-like	28	gi|743787774	3.44
Oil palm profilin-like allergen PF2	14	gi|34223519	3.38
Chaperone protein ClpB1	101	gi|743756256	3.35*
Glycine-rich RNA-binding protein	16	gi|648174145	3.19
Hypersensitive-induced response protein 1 X1	34	gi|743798946	2.8
Leucine-rich repeat extensin-like protein 2	70	gi|743772323	2.52
Universal stress protein A-like protein	24	gi|743845102	2.16
GTP-binding nuclear protein Ran1B-like	25	gi|743769294	2.10
Pathogenesis-related protein PRB1-2-like	23	gi|743761748	2.07
Patellin-3-like	61	gi|743764635	1.97
Aspartic proteinase in guard cell 1-like	48	gi|743766057	1.86
17.4 kDa class III heat shock protein	18	gi|743774135	1.80
Germin-like protein 5–1	24	gi|743762743	1.79
Aldo-keto reductase 2	38	gi|743814040	1.46
Formate dehydrogenase, mitochondrial	41	gi|743838587	1.41
Peroxidase 3-like	35	gi|743820630	1.29
Remorin-like	21	gi|743866636	1.19
26S proteasome non-ATPase regulatory subunit 2 homolog A-like	98	gi|743776123	1.12
Profilin-2-like	14	gi|743795378	-1.01
Protein IN2-1 homolog B-like	28	gi|743892338	-1.13
Protein DJ-1 homolog D-like	41	gi|743834058	-1.22
Universal stress protein A-like protein	19	gi|743784546	-1.28
Heat shock protein 81-1-like	80	gi|743807690	-1.44
Enoyl-[acyl-carrier-protein] reductase [NADH] 1, chloroplastic	40	gi|743808818	-1.59
Serine carboxypeptidase-like	59	gi|743780890	-1.62
Skin secretory protein xP2-like	17	gi|743886010	-1.68
Subtilisin-like protease	81	gi|743774266	-1.78
Subtilisin-like protease	82	gi|743778980	-1.85
Peroxidase 4-like	35	gi|743818796	-2.03
Hypersensitive-induced response protein 1	31	gi|743755088	-2.01
Guanine nucleotide-binding protein subunit beta-like protein A	36	gi|743794305	-2.19
Cationic peroxidase SPC4-like	38	gi|743817476	-2.19
Superoxide dismutase [Cu-Zn], chloroplastic	23	gi|743852970	-2.7
Proteasome subunit alpha type-7	27	gi|743855249	-2.74
Aminopeptidase M1-like	101	gi|743769768	-2.78
Chaperonin CPN60-2, mitochondrial-like	61	gi|743826168	-3.54
Uncharacterized protein YDL057W isoform X1	32	gi|743797589	-3.58
Apyrase 2	50	gi|743789264	-5.25
Uncharacterized protein LOC105034060	28	gi|743875660	-6.16
Isoflavone reductase-like protein isoform X1	34	gi|743871605	Asy^d^*
Isoflavone reductase-like protein	28	gi|743871643	Asy.*
Oryzain alpha chain-like	51	gi|743822079	Asy.
BAG family molecular chaperone regulator 7	48	gi|743872681	Asy.
Tuliposide A-converting enzyme 2, chloroplastic-like	35	gi|743860456	Asy.*
Caffeic acid 3-O-methyltransferase-like	40	gi|743832206	Asy.*
Cinnamyl alcohol dehydrogenase 2-like	39	gi|743861182	Asy.*
Annexin D2-like	36	gi|743801141	Asy.*
Annexin D2-like isoform X2	36	gi|743773114	Asy.*
Subtilisin-like protease SDD1	82	gi|743874008	Asy.*
Subtilisin-like protease SDD1	82	gi|743874008	Asy.*
Beta-galactosidase 15 isoform X1	99	gi|743813552	Asy.
S-adenosylmethionine synthase	43	gi|743783184	Asy.*
Profilin-1-like	14	gi|743799588	Asy.
16.9 kDa class I heat shock protein 2-like	18	gi|743772279	Asy.*
Linoleate 9S-lipoxygenase 5	99	gi|743830998	Asy.*
Allene oxide synthase 2-like	54	gi|743767001	Asy.*
Nudix hydrolase 3 isoform X1	81	gi|743767540	Asy.
UDP-glucuronic acid decarboxylase 6 isoform X1	38	gi|743874409	Asy.*
Dihydroxy-acid dehydratase, chloroplastic	67	gi|743852824	Asy.
Membrane steroid-binding protein 2	29	gi|743776234	Asy.*
Leucine-rich repeat extensin-like protein 5	71	gi|743802043	Asy.
Cysteine synthase	34	gi|743774724	Asy.*
Syntaxin-71-like isoform X2	30	gi|743795463	Asy.
Protein phosphatase 2C 62 isoform X1	31	gi|743758875	Asy.
17.3 kDa class II heat shock protein-like	17	gi|743799089	Asy.*
Bifunctional aspartate aminotransferase and glutamate/aspartate-prephenate aminotransferase-like isoform X2	44	gi|743763413	Asy.
Proteasome subunit alpha type-4	27	gi|743873727	Asy.
Transketolase, chloroplastic	81	gi|743854750	Asy.*
Glutathione S-transferase omega-like 2	39	gi|743771606	Asy.
Peroxidase 15-like	35	gi|743839458	Asy.
Thioredoxin reductase NTRB	39	gi|743821878	Asy.
Glutathione S-transferase 3	24	gi|743844790	Asy.
Peroxidase 72-like	36	gi|743838248	Asy.
Thioredoxin H1	13	gi|743759544	Asy.*
Uncharacterized protein LOC105042730	59	gi|743775881	Asy.
Peroxidase 3-like	36	gi|743768213	Asy.*

^a^MW = Molecular weight.

^b^Log_2_ FC = Log_2_ fold change.

^c^FY = found exclusively in plants with FY symptoms.

^d^Asy. = found exclusively in asymptomatic plants.

* Statistically significant at *p* <0.05 available in S2A, S3A and S4A Tables.

### 3.3. Energy metabolism

Overall, proteins related to energy metabolism presented greater accumulation in asymptomatic oil palm roots of the two sampling areas. Most of these proteins are involved in carbohydrate metabolism, mainly in glycolysis (Tables 3 and 4). Noteworthy, enzymes involved in energy metabolism under anaerobic conditions were also identified.

**Table 3:** Differentially abundant proteins related to energy production in roots of plants of area I with log_2_ fold change ≥ 1 or ≤ -1.

Protein name	MW^a^ (kDa)	Accession	Log_2_ FC^b^
Pyruvate kinase, cytosolic isozyme	41	gi|743816338	1.52
Glyceraldehyde-3-phosphate dehydrogenase GAPCP1, chloroplastic-like	57	gi|743855918	1.51
Fructose-bisphosphate aldolase 1, chloroplastic-like	42	gi|743800712	-1.17
Pyruvate kinase, cytosolic isozyme	46	gi|743852444	-2.32
D-3-phosphoglycerate dehydrogenase 1, chloroplastic-like	65	gi|743809510	Asy^c^.
2,3-bisphosphoglycerate-independent phosphoglycerate mutase-like	61	gi|743843487	Asy.
2-isopropylmalate synthase A-like	68	gi|743821259	Asy.*
Aldehyde dehydrogenase family 2 member B7, mitochondrial-like	62	gi|743767790	Asy.*

^a^MW = Molecular weight.

^b^Log_2_ FC = Log_2_ fold change.

^c^Asy. = found exclusively in asymptomatic plants.

* Statistically significant at *p* <0.05 available in S2A, S3A and S4A Tables.

**Table 4:** Differentially abundant proteins related to energy production in roots of plants of area II with log_2_ fold change ≥ 1 or ≤ -1.

Protein name	MW^a^ (kDa)	Accession	Log_2_ FC^b^
Pyruvate dehydrogenase E1 component subunit beta-1, mitochondrial-like	68	gi|743880374	FY^c^*
Succinate-semialdehyde dehydrogenase, mitochondrial isoform X2	54	gi|743768016	FY
Methylmalonate-semialdehyde dehydrogenase [acylating], mitochondrial isoform X1	57	gi|743759731	FY
V-type proton ATPase catalytic subunit A	68	gi|743809830	3.32
Uncharacterized oxidoreductase At4g09670-like	40	gi|743773279	1.34
Pyruvate dehydrogenase E1 component subunit alpha-1, mitochondrial-like isoform X2	46	gi|743811207	1.22
Aldehyde dehydrogenase family 2 member B7, mitochondrial-like	62	gi|743767790	-1.16
Isocitrate dehydrogenase [NADP]	47	gi|743755796	-1.95
Bifunctional methylthioribulose-1-phosphate dehydratase/enolase-phosphatase E1	57	gi|743876340	-2.25
Dihydrolipoyl dehydrogenase, mitochondrial-like	57	gi|743855576	-2.26*
2-isopropylmalate synthase A-like	55	gi|743821259	-2.74
Pyruvate kinase, cytosolic isozyme	68	gi|743775291	-4.2
UDP-sugar pyrophosphorylase	55	gi|743804515	Asy.^d^
6-phosphogluconate dehydrogenase, decarboxylating 1-like	54	gi|743826796	Asy.
Cytochrome b5-like	15	gi|743765463	Asy.
V-type proton ATPase subunit B 2-like isoform X2	54	gi|743797544	Asy.*
V-type proton ATPase subunit G-like	12	gi|743757417	Asy.
Uncharacterized protein LOC105037637	45	gi|743892552	Asy.*

^a^MW = Molecular weight.

^b^Log_2_ FC = Log_2_ fold change.

^c^FY = found exclusively in plants with FY symptoms

^d^Asy. = found exclusively in asymptomatic plants.

* Statistically significant at *p* <0.05 available in S2A, S3A and S4A Tables.

Still regarding proteins involved in the anaerobic metabolism, a sequence (gi|743767790) of the enzyme aldehyde dehydrogenase was identified in plant samples from the two growing areas with greater accumulation in asymptomatic plants, as shown in Tables 3 and 4. Moreover, alcohol dehydrogenases were also detected in the proteomes of plants from both areas, with high intensity in all analyzed samples (S2 and S3 Tables).

### 3.4. Protein profiling of different stages of FY symptoms

Root proteomes of three different stages of plants afflicted with FY revealed 367 proteins, which were grouped in 197 clusters. As expected, the majority of the detected proteins are related to stress, defense and energy metabolism, including processes such as transport, signalization and oxi-reduction.

**Fig 2:**
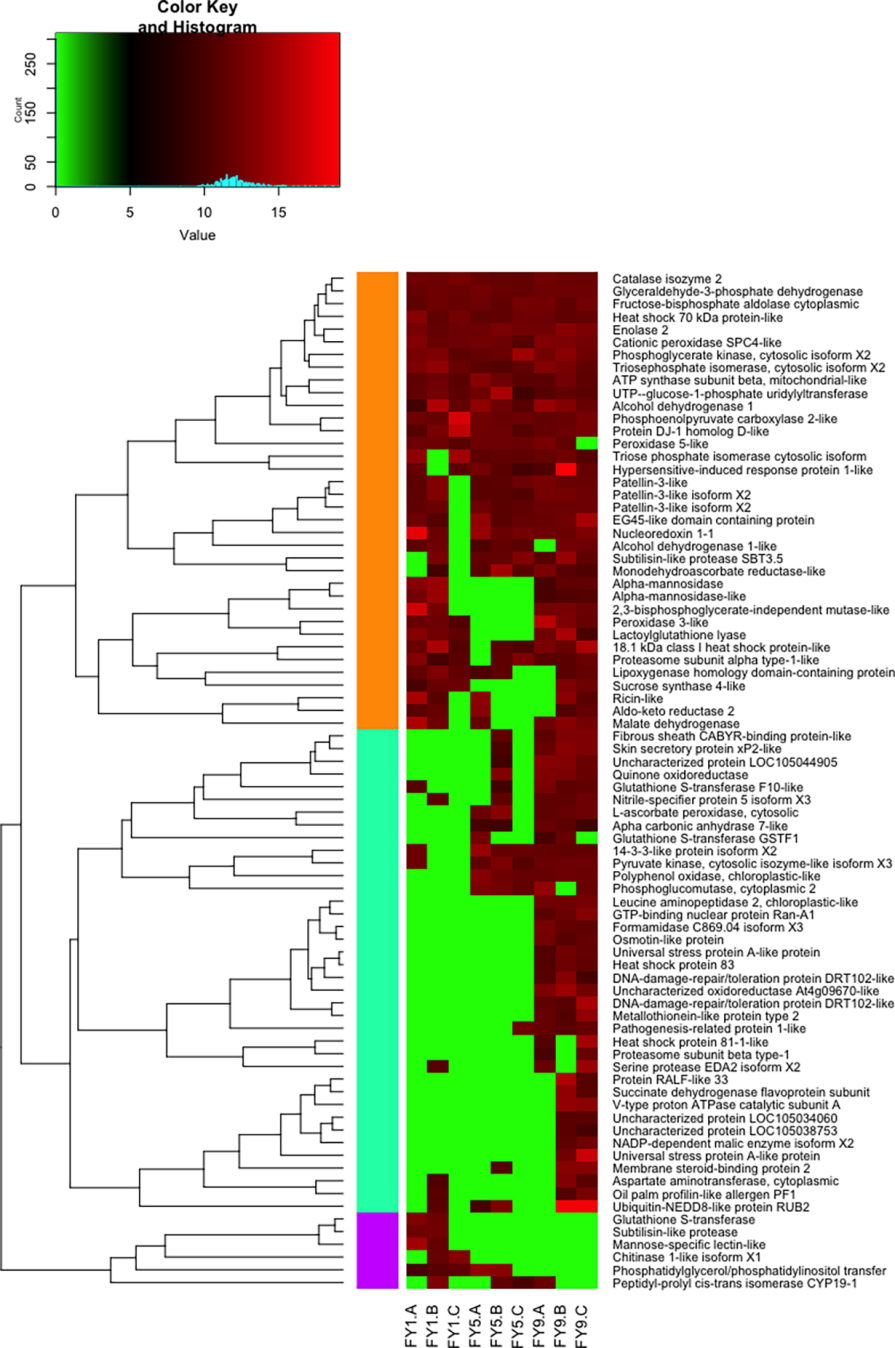
Hierarchical grouping of differentially expressed proteins related to stress response and energy metabolism in oil palm roots at three stages of severity of FY symptoms. Three analytical replicates were analyzed for each stage: initial (FY1.A, FY1.B and FY1.C), intermediate (FY5.A, FY5.B and FY5.C) and advanced (FY9.A, FY9.B and FY9.C).

A hierarchical grouping based on protein accumulation profiles involved in response to stress and energy metabolism at different stages of symptom severity revealed the formation of three groups indicated by orange, green and purple bars, respectively (Fig 2). The first group (orange bar) consisted mainly of proteins that showed high accumulation in the three stages of severity of FY symptoms. Noteworthy are those involved in the antioxidant system and the metabolism of carbohydrates. In addition, sequences of alcohol dehydrogenase were identified with high intensity from the onset of symptoms, and were grouped together with other proteins involved in energy production. The second group (green bar) included proteins with high accumulation, mainly in the most advanced stage of FY severity (FY9). In this group, we observed proteins related to biotic stress response and ROS homeostasis. The third group (purple bar) consisted of a small number of proteins identified only in the initial (FY1) or intermediate (FY5) stages. This group consisted of only six proteins, with the majority being related to the response to biotic and abiotic stresses.

## 4. Discussion

### 4.1. Stress-related proteins and their possible relationships with FY

The lower accumulation of stress-responsive proteins in plants with fatal yellowing symptoms and the identification of some of these proteins only in asymptomatic plants suggest that the two cultivated fields may be in areas susceptible to FY development, but asymptomatic plants still exhibited greater resistance to a stress, which could be related to FY development. In this context, proteins identified in this study may be fundamental to the mechanism of resistance to FY.

Among them we identified proteins involved in phenylpropanoid and lignin metabolism to be down-regulated in plants with FY symptoms. Transketolase and isoflavone reductase are involved in phenylpropanoid synthesis, which are important to stress response [26, 27]. Henkes et al. (2001) [26] verified that partial inhibition in transketolase activity resulted in decreased production of phenylpropanoid metabolism products like lignin, as well as chlorophyll and carotene, in tobacco. Isoflavone reductase is essential for the response to various biotic and abiotic stresses [27], by participating in the synthesis of phytoalexins that help increase tolerance in plants submitted to different environmental stresses. The decrease in phenylpropanoid biosynthesis can be considered one of the determining factors for aggravation of FY symptoms. Down-regulation of those enzymes, as found in this study, can be determinant of the FY development in symptomatic plants.

Concerning lignin metabolism, the enzymes cinnamyl alcohol dehydrogenase and caffeic acid 3-O-methyltransferase were also identified only in asymptomatic plants. These enzymes participate in the biosynthetic pathway of lignin. Caffeic acid 3-O-methyltransferase belongs to a family of methyltransferases that are dependent on S-adenosylmethionine. One sequence related to S-adenosylmethionine synthase was also identified only in asymptomatic plants. This enzyme also plays a crucial role in methionine biosynthetic metabolism, besides protein biosynthesis. The up-regulation of S-adenosylmethionine synthase in plants has been related to biotic and abiotic stress responses, like those caused by herbivore insects and salt stress [28].

Lignin is the second most abundant biopolymer in plants after cellulose and it is essential for the structural rigidity, and defense mechanism of plants, acting as a physical barrier to pathogen attack [29]. Thus, the identification of caffeic acid 3-O-methyltransferase, S-adenosylmethionine synthase and cinnamyl alcohol dehydrogenase only in roots of asymptomatic plants suggests that root lignification is a fundamental process against aggravation of FY symptoms, promoting greater resistance against the appearance of necroses already seen in roots of plants with FY [14] and dissemination of opportunistic pathogens in roots of weakened plants.

Other proteins involved in the response to pathogen attacks are the sieve occlusion elements (SEO), which act in phloem tubes for immediate sealing after injuries. They have also been found to be efficient in avoiding photoassimilate loss and pathogen diffusion by the phloem [30]. Froelich et al. (2011) [31] performed studies with these this protein class in *A*. *thaliana* and reported its positive effects on defense against pathogens, in addition to not showing any obstruction of sieve. On the other hand, there is a possibility that agglomerate formation may obstruct the sieve translocation [30], which impairs the mass fluxes inside the plant. Recently, Srivastava et al. (2016) [32] also observed that the expression of these proteins increases the tolerance to salinity stress in tobacco. In this way, this class of proteins does not only act in response to biotic stress. However, more research is necessary to understand the enhanced accumulation of these proteins in FY conditions in oil palms.

Considering plants with symptoms at different stages of FY (initial, intermediate and advanced), proteins related to biotic stresses were more abundant in roots with more advanced symptoms (Fig 2), which is consistent with the hypothesis of increased infection by opportunistic pathogens. Therefore, even though a wide variety of stress response related proteins were identified in asymptomatic plants, this does not imply that the development of FY symptoms is initiated by a biotic agent. However, the hypothesis that FY has its initial symptoms occasioned by a biotic agent should not be ruled out.

### 4.2. Proteins associated with antioxidation and detoxification

FY is largely an unknown syndrome or pathology in oil palms andscarce physiological evidences are available to understand this species’ behavior in environments subject to flooding or waterlogged soil. The FY symptoms have been associated with soil compaction, resulting in lower hydraulic conductivity and porosity. This fact has already been observed in clayey soils containing oil palm crops with high FY incidence [33, 6, 34].

In waterlogged soils, the absorption of nutrients like Ca and P is impaired, and some elements like Fe and Mn can be reduced to more soluble forms and reach toxic levels to the plant [35, 11, 14, 36, 37]. Concomitantly, these effects can trigger oxidative stress [35, 38]. This effect can be enhanced when flooding and post flooding are combined with high irradiance and temperature, as it regularly occurs in the Amazon Region.

Proteins related to the antioxidant system had greater accumulation in asymptomatic plants in the two oil palm growing areas (Tables 1 and 2). The formation of reactive oxygen species is related to the regulation of signaling pathways and initial responses to various environmental stresses, including excessive accumulation of essential elements. Concomitantly, the antioxidant system acts for O_2_ and H_2_O_2_ elimination in different subcellular compartments [39, 40, 20]. These mechanisms have been shown to be fundamental for the resistance to oxidative stress such as that caused by soil flooding, based on previous studies carried out with proteomic analyses in crops submitted to this stress [19, 20, 21].

Still regarding protection against oxidative stress, ferritin is an important protein for plants’ detoxification. In plants, ferritins are present in the plastids and are involved in Fe transport and storage due to their high affinity and capacity to accommodate this element [41]. The increase in ferritin expression also occurs in response to oxidative stress, since the excess of Fe generates reactive oxygen species through the Haber-Weiss reaction [42, 43]. Increased levels of free Fe in cell is toxic, and the ferritin acts as a detoxification protein [36, 38, 44]. In this study, iron concentrations in the plants were not verified. However, it is common to observe an increase in the concentration of iron in plants submitted to hypoxic environments such as flooded soils [45, 43, 46].

Proteomic [47] and gene expression [48] assays reported accumulation of ferritin in plants submitted to flooding, suggesting that this protein plays a significant role in the defense of plants against oxidative stress in this condition.

Ferritin was identified only in asymptomatic plants of area I. Knowing the significant role of ferritin in iron homeostasis in plant cells, its detoxification could be an essential process in oil palm resistance to root structural weakness and greater tolerance to FY development. This enzyme together with other components of the antioxidant system can be considered as an important factor to the tolerance of FY in response to flooded or waterlogged soils.

Asymptomatic plants showed greater accumulation of proteins related to biotic and abiotic stress compared with plants showing symptoms. The synergic action of stress related-proteins in asymptomatic plants can enable higher tolerance to FY development in these genotypes.

Asymptomatic plants may comprise genotypes with adaptations that allow greater tolerance to different environmental stresses, biotic or abiotic. This is due to a more effective response, including expression of genes for the synthesis of proteins involved in mechanisms related to plant tolerance to stresses such as flooding, insect and pathogen defense, and response to oxidative stress that may occur in a variety of situations. However, few studies have been conducted in oil palm to identify *Elaeis guineensis* varieties with different genotypes or with different degrees of tolerance to FY. In a study of genetic characterization, Costa et al. (2014) [49] compared RAPD (Random Amplification polymorphic DNA) with a population of 51 oil palm plants also from Mojú, Pará State, Brazil, where 24 plants had the symptoms of FY and 27 were apparently healthy. The authors did not observe significant differences to genetically discriminate within groups of affected plants and healthy ones, not being able to attribute a genetic cause to FY.

Scientific advances have allowed the use of genome-wide molecular markers for the identification of genetic alterations, allowing the discrimination of genotypes (cultivars or varieties) of the same species with tolerance-related characteristics to several environmental stresses [50, 51, 52, 53, 54, 55]. Studies with this scope have given subsidies to plant breeding programs [56, 57], but genetic variability in oil palm individuals with and without FY symptoms still deserve further investigation.

### 4.3. Proteins involved in energy and fermentative metabolism

Components of the primary metabolism are involved in defense in plants [58]. Energy production is fundamental for the expression of genes for the biosynthesis of proteins involved in stress response mechanisms [59]. A positive regulation of transcripts involved in increased energy metabolism has been observed in response to biotic and abiotic stress [60]. However, in contrast to the high levels of energy metabolism proteins since the initial stage of the FY, proteins related to defense mechanisms were more abundant only in the advanced stages of the symptoms.

Additionally, we also identified high intensity of alcohol dehydrogenase in plant roots with and without the symptoms of the FY in the oil palms in both growing areas, and in the proteomes at all three stages of FY severity. Alcohol dehydrogenases are involved in alcoholic fermentation, where they catalyze the conversion of pyruvate to ethanol. During oxygen deficiency, there is a decrease in energy production through oxidative phosphorylation, and the fermentative metabolism promotes energy compensation through the recycling of NAD^+^ to the glycolytic pathway [61, 62]. Therefore, alcohol dehydrogenase plays a fundamental role in the maintenance of energy metabolism under anaerobic conditions.

The increase of proteins of the glycolytic pathway and involved in the anaerobic respiration related to the alcohol fermentation process has been identified as a key response of plants to hypoxia in flooded soils [19, 63]. In our results, aldehyde dehydrogenase was more accumulated in asymptomatic plants. Under anaerobic conditions, amounts of acetaldehyde are produced which can cause toxicity to the plant. At this time, the aldehyde dehydrogenase converts the alcetaldehyde to acetate. Thus, this enzyme has been related to higher plant survival under anaerobic conditions [64, 65, 66, 67, 68].

Overexpression of the ADH1 gene had no effect on flood tolerance in Arabidopsis, but its levels of expression under anaerobic conditions were found to be critical for plant survival under anaerobic conditions [62]. Moreover, Bertolde et al. (2014) [40] showed that in flooded *Theobroma cacao* tolerant genotype the ADH gene was overexpressed in comparison to the susceptible genotype. Thus ADH levels in oil palms with and without FY symptoms may not be a critical point for tolerance to the development of symptoms, but an indicator that these plants have been exposed to anaerobiosis.

Furthermore, there may be temporal differences in ADH expression between tolerant plants and more susceptible to hypoxia or anoxia conditions, as well as between different plant species, which would influence response mechanisms for anaerobic survival [69]. Therefore, it is important to emphasize that in this study, plants were analyzed under field conditions and it is not possible to infer the exact moment when plants were submitted to such stress. Thus, the difference between ADH expression “response-time” within plants with and without FY symptoms could also be considered as a factor influencing FY development.

The adaptation to hypoxia or anoxia includes metabolic alterations such as the synthesis of anaerobic stress proteins (ANPs), which includes enzymes of glycolysis, ethanol fermentation and carbohydrate metabolism [16, 70]. In addition to the enzymes already mentioned, other enzymes essential to carbohydrate metabolism were identified, mostly down-regulated in plants with symptoms, as can be seen in Tables 3 and 4. These enzymes were also found in plant roots at different stages of development of FY symptoms, as shown in Fig 2.

The relationship between the higher incidence of FY, increase in rainfall, soil flooding index [10, 12, 13] high levels of glycolytic pathway enzymes and the presence of aldehyde dehydrogenase, alcohol dehydrogenase and many antioxidant enzymes, suggests that the plants from the two areas of oil palm cultivation may have been submitted to a recent or constant state of hypoxia in the roots.

The higher accumulation of proteins involved in energy production, including aldehyde dehydrogenase, in asymptomatic plants, may also be associated with a more efficient response of those genotypes against FY development, through more efficient use of energy under anaerobic conditions.

## 5. Conclusions

This was the first study aiming to describe protein alterations associated with FY in oil palm roots. Protein analysis proved to be a powerful tool to shed light on the molecular mechanisms related to the tolerance and development of FY disease. Putative protein markers that could to guide selection of FY-tolerant oil palm genotypes were also identified.

Proteins related to anaerobic metabolism were found in all sampled plants, whether symptomatic or not, suggesting a recent or constant condition of hypoxia in their respective environments. Comparing different stages of FY symptoms’ severity, the higher intensity of alcohol dehydrogenase and energy related-proteins since the onset of symptoms contrasted with the increase of biotic stress related proteins in later stages of the syndrome. Our finding suggests that changes in abiotic factors may precede the occurrence of FY, paving the way for opportunistic pathogens.

## Supporting information

S1 Table Steps and procedures of protein extraction according with SDS/Phenol protocol proposed by Wang (2006) [24] with some modifications. (PDF)

S2 Table Data from Marborges (Area I) plant proteomes. **A**) Quantitative samples view. **B**) Protein report. **C**) Quantitative peptide report. **D**) Quantitative spectrum report.
(XLS)

S3 Table Data from Biopalma (Area II) plant proteomes. **A**) Quantitative samples view. **B**) Protein report. **C**) Quantitative peptide report. **D**) Quantitative spectrum report.
(XLS)

S4 Table Data from Marborges (Area I) plant proteomes for analysis of plants between different stages of fatal yellowing symptoms. **A**) Quantitative samples view. **B**) Protein report. **C**) Quantitative peptide report. **D**) Quantitative spectrum report.
(XLS)

S1 Highlights Manuscript highlights. (DOC)
