# Supplementary material for: Differential accumulation of proteins in oil palms affected by fatal yellowing disease
Source: PLoS One. 2018 Apr 5;13(4):e0195538. doi: 10.1371/journal.pone.0195538 (PMC5886584; doi:10.1371/journal.pone.0195538)
Supplement: S1 Table — (PDF) [file pone.0195538.s001.pdf]

Differential accumulation of proteins in oil palms affected by fatal yellowing disease

S1 Table Steps and procedures of protein extration according with SDS/Phenol protocol proposed by Wang (2006) [24]with some modifications.

Steps	Procedure
1	The roots of five plants were mixed for each condition and the material was macerated in liquid nitrogen. Were using about 300 mg of macerated roots.
2	In each sample were added 10 mL of buffer containing sucrose (1.5 M), Tris-Hydrochloride (1M, pH 8), sodium dodecyl sulfate (SDS, 10%), (Phenylmethylsulfonyl fluoride (PMSF, 100 mM), polyvinylpolypyrrolidone (PVPP) and ultrapure water with addition of 100 µL of protease inhibitor (SIGMA) and 500 µL of β-mercaptoethanol.
3	The samples were sonicated for five repetitions of 30 seconds at room temperature.
4	The extracts were divided in ten microtubules each, followed by the addition of 700 µL of phenol per microtubule. The samples were vortexed by 15 seconds and centrifuged during 8 minutes at 14000 rpm, for the phenolic phase separation.
5	The phenolic content was transferred to new microtubule and the operation repeated to eliminate the any residue of aqueous phase or SDS.
6	There were added 1300 µL of ammonia acetate (100 mM) in methanol (100%) for protein precipitation during 24 hours at -80 ºC. After this time, a centrifugation at 14000 rpm during 8 minutes, and the supernatant was discarded.
7	Centrifugation at 14000 rpm during 8 minutes, and the supernatant was discarded. The precipitate was transferred to a new microtubule and washed four times with acetone 80%.
8	The last washing was made with ethanol 70% and the precipitate dried at room temperature in vacuum concentrator around seven minutes.
9	The extracts were solubilized in 200 µL of urea buffer (7 M) and tioureia (2 M).
