# Supplementary material for: Differential accumulation of proteins in oil palms affected by fatal yellowing disease
Source: PLoS One. 2018 Apr 5;13(4):e0195538. doi: 10.1371/journal.pone.0195538 (PMC5886584; doi:10.1371/journal.pone.0195538)
Supplement: S1 Highlights — (DOC) [file pone.0195538.s005.doc]

**Highlights**

- Comparative proteomics revealed changes in proteins related to stress responses and energy metabolism
- Proteins related to anaerobic metabolism were found in high quantities in roots of all sampled plants, suggesting a recent or constant condition of hypoxia in their respective environments.
- Same group of proteins were found to be accumulated since the onset of Fatal Yellowing symptoms meanwhile biological stress and pathogenesis-related proteins were more accumulated at later stages of the disease.
- This findings supports the hypothesis that changes in abiotic factors may precede the occurrence of Fatal Yellowing, paving the way for opportunistic pathogens.
